# Contrast-enhanced mammography in low- and middle-income countries: opportunities and challenges

**DOI:** 10.1097/MS9.0000000000005259

**Published:** 2026-06-11

**Authors:** Hafiza Qurat Ul Ain, Muhammad Junaid Tahir, Khabab Abbasher Hussien Mohamed Ahmed, Waleed Tariq, Aamer Iftikhar

**Affiliations:** a Combined Military Hospital (CMH) Multan, Multan, Pakistan; b Shaukat Khanum Memorial Cancer Hospital and Research Centre, Lahore, Pakistan; c Faculty of Medicine, University of Khartoum, Khartoum, Sudan; d Mayo Hospital, Lahore, Pakistan

Breast cancer remains the most common malignancy and a leading cause of mortality among women globally^[^1^]^. The burden is disproportionately higher in low- and middle-income countries (LMICs), where diagnostic delays and limited imaging infrastructure are prevalent^[^2^]^. Contrast-enhanced mammography (CEM) has emerged as a promising imaging technique that combines anatomical and functional assessment, offering a significant advantage, especially in resource-limited settings. Its role in LMICs should be interpreted with caution, as these settings are highly heterogeneous^[^1,3^]^. In this context, Pakistan is presented as a representative case study, but the feasibility and scalability of CEM are context-dependent.

CEM is feasible in several LMICs, including India^[^4^]^ and Brazil^[^5^]^, where it serves as a cost-effective alternative to breast magnetic resonance imaging (MRI). However, high equipment and contrast costs continue to limit its wider adoption. Affordable, accessible, and accurate imaging technologies are therefore essential for improving outcomes. Among available modalities, CEM has emerged as a promising innovation for LMICs. It integrates dual-energy digital mammography with intravenous iodinated contrast injection to visualize vascular enhancement and tumor morphology^[^6^]^. This technique allows simultaneous anatomical and functional assessment, resembling the angiographic insights of breast MRI. Studies have demonstrated diagnostic sensitivities exceeding 90% for invasive carcinomas, with results comparable to MRI in both detection and characterization of lesions. CEM examinations are performed in approximately 7–10 minutes, significantly shorter than MRI protocols that may extend up to 45 minutes^[^7^]^. The method uses standard mammography units with software-based dual-energy upgrades, reducing the need for specialized hardware. Its ability to provide high-resolution images with minimal infrastructural investment makes it highly suitable for LMIC settings.

Breast MRI remains the reference standard for evaluating breast malignancy, especially for screening high-risk women and preoperative staging^[^8^]^. However, MRI demands high capital costs, specialized coils, and expert interpretation. In LMICs, these requirements are often unaffordable and geographically inaccessible. At Aga Khan Hospital, where CEM was performed as an initiative in Pakistan, it is claimed that CEM costs less than a fifth of the cost of an MRI^[^9^]^. An analysis of U.S. Medicare data estimated the cost of a CEM-guided biopsy and post-clip mammography at $475.48, compared to $845.94 for an MRI-guided biopsy, highlighting the greater affordability of CEM-based procedures^[^10^]^. Initial research also shows that CEM-guided biopsy is a feasible alternative method and a comparatively faster solution for MRI-only detected enhancing lesions^[^11^]^.

Evidence shows that CEM has a diagnostic performance comparable to MRI, particularly in detecting multifocal, multicentric, and contralateral disease^[^10^]^. Furthermore, CEM is well tolerated by patients who experience claustrophobia or possess metallic implants contraindicated by MRI. Comparative studies report no statistically significant difference in sensitivity between CEM and MRI for invasive cancer detection. Thus, CEM offers a clinically robust yet economically viable alternative, especially for LMIC populations^[^9^]^. However, the comparative efficacy of CEM is not uniform across all applications. The efficacy of CEM may vary in situations such as screening high-risk populations, evaluating non-mass augmentation, and assessing treatment response, wherein MRI remains the gold standard^[^12^]^. Consequently, CEM should be seen as a clinically valuable complementary or alternative approach in specific settings rather than a universal replacement for MRI.

Implementing CEM across LMICs requires a structured evaluation. Initially, tertiary care hospitals should conduct pilot studies to assess image quality, diagnostic accuracy, and workflow efficiency compared with MRI^[^2^]^. Performance metrics should include sensitivity, specificity, lesion characterization, and time-to-report parameters^[^1^]^. In subsequent phases, multicenter collaborations involving regional hospitals and teleradiology networks can assess real-world feasibility. Patient selection criteria should prioritize women with dense breasts, inconclusive mammograms, or contraindications to MRI^[^8^]^. These steps will generate localized evidence guiding national imaging policies. CEM demonstrated substantial diagnostic concordance with MRI in most cases, with significant benefits in cost reduction and workflow efficiency. Targeted CEM integration may support more sustainable, value-based breast imaging without compromising diagnostic quality^[^12^]^. Despite the lower operational cost and shorter examination time of CEM, economic evaluations, including cost–benefit and cost–utility analyses within specific healthcare settings, are essential before broader conclusions regarding scalability and affordability can be drawn.

Pakistan faces an uneven imaging infrastructure, concentrated primarily in metropolitan centers. Many secondary hospitals possess digital mammography systems that can be upgraded to perform CEM^[^2^]^. The practical implementation of CEM requires several key components: (1) upgrading existing digital mammography systems to support dual-energy imaging, (2) ensuring reliable procurement and supply chains for nonionic iodinated contrast, (3) structured training programs for radiologists and technologists in contrast administration, image acquisition, and interpretation of the imaging, (4) standard screening protocols for renal function assessment and management of contrast-related adverse reactions, and (5) integration into existing diagnostic and referral pathways, including linkage of CEM images with Picture Archiving and Communication Systems (PACS), enabling remote consultations and teleradiology. These operational requirements are necessary for the safe, effective, and sustainable adoption of CEM in LMICs.

In LMICs, cost-effectiveness and accessibility are critical. Pakistan’s annual per capita healthcare expenditure remains below USD 50, limiting access to advanced diagnostics. CEM addresses these constraints by offering high-quality imaging at reduced cost and shorter examination times. High-throughput potential allows more patients to be screened daily, optimizing resource utilization^[^7^]^. Integrating CEM into breast cancer screening programs could improve early detection and survival outcomes, especially among women with dense breasts or high familial risk^[^13^]^. This technology could also reduce unnecessary referrals to tertiary MRI centers, improving equity and reducing the overall system burden.

Despite its advantages, CEM has some inherent limitations. Radiation exposure is approximately 20–30% higher than conventional mammography but remains within acceptable safety thresholds established by the American College of Radiology^[^13^]^. The use of iodinated contrast introduces potential risks of hypersensitivity or nephrotoxicity, necessitating pre-procedural assessment and emergency readiness. Moreover, the ability of CEM to detect microcalcifications is slightly inferior to standard mammography, warranting combined interpretation. The variability in infrastructure and workplace capacity across LMICs, potential challenges in the provision of iodinated contrast, and limited availability of standardized training programs should be considered. Furthermore, the majority of existing evidence is derived from single-center or high-resource settings, restricting generalizability. Diverse data from larger and multicenter studies are needed to assess the role of CEM in routine clinical practice.

CEM is an innovative, affordable, and technically feasible imaging modality capable of improving breast cancer diagnostics in LMICs. Its shorter examination time, high diagnostic accuracy, and compatibility with existing equipment make it an ideal alternative to MRI in resource-limited healthcare systems. For Pakistan, integrating CEM through phased pilot projects, standardized protocols, and national screening programs could markedly enhance early detection and treatment outcomes. To establish CEM in Pakistan, upgraded mammography systems compatible with contrast imaging are needed, as most existing units cannot perform CEM. Additionally, structured training programs for radiologists, technologists, and trainees are essential to ensure the safe and effective utilization of this technique. By bridging the gap between cost and clinical sophistication, CEM can significantly contribute to reducing the burden of breast cancer in LMICs.

## Conclusion

- CEM offers diagnostic performance comparable to breast MRI for detecting and characterizing invasive breast cancer, while requiring substantially lower costs, shorter examination times, and less specialized infrastructure – making it particularly suitable for LMICs.
- CEM can be implemented in resource-limited settings using upgraded digital mammography systems, enabling both anatomical and functional breast assessment without the need for dedicated MRI facilities and improving access to advanced breast imaging where MRI availability is limited.
- Integrating CEM into LMIC breast cancer diagnostic pathways has the potential to enhance early detection, optimize healthcare resource utilization, and reduce diagnostic delays, provided that standardized training, contrast safety protocols, and phased implementation strategies are established.

## Data Availability

Data availability is not applicable to this article, as no new data were created or analyzed in this study.
